# Radiotherapy in Oligometastatic, Oligorecurrent and Oligoprogressive Prostate Cancer: A Mini-Review

**DOI:** 10.3389/fonc.2022.932637

**Published:** 2022-06-08

**Authors:** Alexander Yaney, Andrew Stevens, Paul Monk, Douglas Martin, Dayssy A. Diaz, Shang-Jui Wang

**Affiliations:** ^1^Department of Radiation Oncology, The Ohio State University Wexner Medical Center, Columbus, OH, United States; ^2^College of Medicine, The Ohio State University, Columbus, OH, United States; ^3^Medical Oncology, The Ohio State University Wexner Medical Center, Columbus, OH, United States

**Keywords:** prostate cancer, radiotherapy, oligometastatic prostate cancer, oligorecurrent prostate cancer, oligoprogressive castration resistant prostate cancer, ADT (androgen deprivation therapy)

## Abstract

Globally, prostate cancer is one of the most common malignancies affecting men. With the advent of advanced molecular imaging, an increasing number of men are found to have oligometastatic disease (OD) either at primary diagnosis or at the time of biochemical failure. No strict definition exists for OD, with historical and ongoing studies utilizing diverse criteria. There is mounting evidence from many different malignancies that patients with OD have improved outcomes compared to their widely metastatic counterparts. As such, treatment intensification of those with OD or oligoprogressive disease has become an area of intense interest and study. This article will review the biology, evidence and controversy behind the treatment of *de novo* oligometastatic, oligorecurrent and oligoprogressive prostate cancer.

## Introduction

Prostate cancer is the second most common malignancy and sixth most common cause of cancer-related death among men worldwide (1). Due to the advent of screening prostate-specific antigen (PSA), prostate cancer is typically diagnosed early in the disease course, particularly in developed countries. However, PSA screening recommendations made by the USPSTF in 2012 led to a decline in PSA screening, which resulted in an increase in the incidence of high-risk and metastatic disease at diagnosis, particularly in certain racial and ethnic groups (2–4). While outcomes for low- and intermediate-risk prostate cancer are favorable, a significant proportion of men with high-risk disease will experience recurrence and spread of their cancer (5, 6).

Of those diagnosed with metastatic disease at any point in their disease course, a wide spectrum in total metastatic burden exists, from a single lesion to diffuse disease. Traditionally, systemic therapy has been the mainstay of treatment for these patients, with radiotherapy being used for palliation, if warranted (7, 8). However, recently this treatment paradigm is shifting, particularly in the setting of oligometastatic, oligorecurrent and oligoprogressive disease (9–11).

The aim of this mini-review is to present and summarize the concepts of oligometastatic, oligorecurrent and oligoprogressive prostate cancer in addition to the current evidence on the role of radiotherapy in the management of these distinct disease entities. Evidence to include in this mini-review was obtained through search of PubMed for peer-reviewed, original studies on prospective trials and clinicaltrials.gov for ongoing/accruing trials in the three distinct oligometastatic disease settings.

## Background and Definitions

While the exact meaning of clinical oligometastatic disease is controversial, most recent clinical trials and clinical reviews have used ≤3-5 metastatic lesions (12). This disease state can be seen either at the time of initial diagnosis (with synchronous metastases), at which point it is considered *de novo* oligometastatic disease, or in the recurrent setting (with metachronous metastases), which is considered oligorecurrent disease (ORD) (12). The exact prevalence of oligometastatic prostate cancer is difficult to describe with any degree of certainty due in part to the lack of a standardized definition, different clinical scenarios in which it can arise (*de novo* or recurrent) and the varying imaging modalities utilized to stage these men (conventional imaging vs. positron emission tomography (PET)-imaging) (10). One study by Larbi et al. utilized whole body MRI to determine the proportion of patients with oligometastatic disease (defined as ≤3 lesions) among 96 men diagnosed with *de novo* metastatic prostate cancer and found that 28% of patients with castration-naïve prostate cancer and 50% of those with castration-resistant disease met criteria for oligometastatic disease (13). Likewise, a study by Müller et al. sought to determine the prevalence of oligometastatic disease (defined as ≤3 lesions) in 110 men with biochemical recurrence after prostatectomy utilizing prostate-specific membrane antigen (PSMA) PET imaging and reported that 30% of patients could be classified as having oligometastatic disease (14).

What is perhaps more important than the clinical definition of oligometastatic disease is its biologic definition and its accompanying implications. Multiple models for the biology of cancer exist, the oldest being the Halsted theory, which proposes that cancer spreads in an orderly fashion from the primary site to regional lymphatics to distant locations (15). In contrast to this, the Fisher theory proposes that cancer is inherently a systemic disease, even if it is evident only locally (16). It is in the third theory of cancer biology, the spectrum theory, in which the concept of oligometastatic disease lies and its importance is highlighted. In this theory, cancer exists in various degrees of clonal evolution, with varying metastatic potential, which evolves over time. In this theory, the concept of oligometastatic disease represents just one timepoint along the evolution of disease, a point which could represent an intermediate state between localized and widely metastatic disease in which cancer cells have limited metastatic potential and thus may be amenable to cure with total elimination of disease burden (12, 17, 18).

The concept of eradication of oligometastatic lesions as a means of improving cancer-specific outcomes has been studied in several malignancies. For instance, surgical resection of liver metastases in addition to primary disease from colorectal cancer confers cure in one of six patients (19). Similarly, local consolidation of primary and oligometastatic sites in both non-small cell lung cancer (NSCLC) and breast cancer have led to significant improvements in or prolongation of progression-free survival (PFS) and overall survival (OS) (19–24). However, management of men with *de novo* or recurrent oligometastatic prostate cancer (OPC) is currently controversial, especially in regard to metastases-directed local therapy. This is reflected by the largely ambiguous guidelines provided in the Prostate Cancer NCCN guidelines, stating that SBRT to metastasis can be considered in the setting of 1) limited metastatic disease when ablation is desired (e.g. impending fracture or encroachment on spinal nerves/vertebrae), 2) in oligometastatic progression when PFS is the primary goal, or 3) if there is a symptomatic lesion in or close to a previously treated region (25). Optimal management of men with *de novo* or recurrent OPC has become more important now than ever due to the advent of advanced molecular imaging, such as PSMA-PET, which has led to an increase in the number of men diagnosed with oligometastatic disease due to its improved sensitivity and specificity over conventional imaging (CI) (26).

On the other hand, oligoprogressive disease is characterized by disease progression in a few sites (again, usually ≤3-5) while on systemic therapy, with the disease biology complicated by selective pressure from systemic treatment. While metastases-directed therapy for both lung and breast cancer in the *de novo* oligometastatic setting appears to be beneficial, recent phase II data suggests that local therapy to oligoprogressive lesions improves outcomes in NSCLC, but not in breast cancer, underscoring the notion that oligometastatic disease is driven by underlying biology rather than a strict clinical definition (27).

The optimal treatment paradigm for oligoprogressive prostate cancer also remains unclear (12). MDT in oligoprogressive prostate cancer is often utilized to delay a change to next-line systemic therapy, although prospective data is lacking to demonstrate outcome benefits of this clinical practice.

## Radiotherapy in *De Novo* Oligometastatic Disease

While available data to guide the use of radiation (RT) in *de novo* OPC is sparse, existing studies sought to address the role of 1) treatment to the primary tumor and 2) treatment to both the primary tumor and oligometastatic sites (see **Table 1**). STAMPEDE Arm H examined the use of radiotherapy to the primary tumor in men with newly diagnosed metastatic prostate cancer. In this study, 2,061 men with metastatic prostate cancer (mPCa) of any metastatic disease burden were randomized to systemic therapy (androgen deprivation therapy (ADT) alone from 01/2013 to 12/2015, with docetaxel allowed in addition to ADT from 12/2015 onward) with or without RT to the prostate to either 36 Gy in 6 fractions given once weekly or 55 Gy in 20 fractions delivered over 4 weeks. While the primary endpoint of OS difference was not significant between the RT and no RT arms, subgroup analysis showed a significant improvement in OS in men who received prostate RT in the setting of low-volume metastatic disease as per the CHAARTED trial (defined as not having visceral metastases or ≥4 bone metastases with at least one outside of the spine/pelvis; HR 0.68, 95% CI 0.52-0.9; p=0.007), indicating a potential benefit of primary site local therapy in the setting of *de novo* OPC. Subsequently, an exploratory analysis of STAMPEDE Arm H using a more refined definition of metastatic disease burden was performed. In this analysis, patients with non-regional lymph node or ≤3 bone metastases were found to have improved OS (HR 0.62, 95% CI 0.46-0.83 vs. 1.08, 95% CI 0.91-1.28, p=0.003, respectively) and failure-free survival (0.57, 95% CI 0.47-0.70 vs. 0.87, 95% CI 0.76-0.99, p=0.002, respectively) compared to those with ≥4 bone or any visceral metastases (9). Furthermore, of 1939 men with skeletal metastases, the benefit of local therapy continuously decreased with increasing number of lesions (28). Importantly, only 5% of patients in the radiotherapy arm reported a grade 3 (G3) or grade 4 (G4) toxicity and no grade 5 (G5) toxicities were noted, indicating that the potential benefit of local therapy in OPC is not offset by significant toxicity (9, 29). Along with showing clinical benefit, local therapy appears to be cost-effective. Lester-Coll et al. conducted a cost-effectiveness study utilizing data from men with low-burden metastatic disease utilizing STAMPEDE Arm H data and found that the inclusion of prostate-directed radiotherapy in addition to ADT was associated with higher quality-adjusted life years at a lower cost than ADT alone, with a savings of >$30,000 with lifetime follow-up (30).

**Table 1:** Prospective trials evaluating the role of radiotherapy in (oligo)metastatic prostate cancer.

Study	Disease Type	Metastatic Burden	Study Type	N	Randomization (if applicable)	Primary Outcome	Result	Toxicity
STAMPEDE Arm H	*De novo* metastatic disease	Any metastatic burden	Phase III RCT	2,061	ADT (+/- docetaxel) vs. ADT (+/- docetaxel) with prostate RT	OS	OS difference not significant overall (HR 0.92, 95% CI 0.80-1.06). Subgroup analysis showed significant benefit in OS for those with low metastatic burden (HR 0.68, 95% CI 0.52-0.9; p=0.007)*	G3 or G4 in 5% of patients
HORRAD	*De novo* metastatic disease	Any metastatic burden	Phase III RCT	432	ADT alone vs. ADT with prostate RT	OS	OS difference not significant (HR=0.90, 95% CI 0.70-1.14)	Not reported
SABR-COMET	ORD	1-5 metastases	Phase II RCT	99**	MDT vs. Standard of care for their respective malignancies	OS	OS improved in MDT arm (5-year OS 42.3% vs. 17.7%, p=0.006)	G5 in 4.5% of patients
STOMP	ORD	≤ 3 metastases	Phase II RCT	62	MDT vs. Observation	ADT-free survival	Median ADT-free survival improved in MDT arm (5-year ADT-free survival 34% vs. 8%, p=0.06)	No G2 or higher
ORIOLE	ORD	≤ 3 metastases	Phase II RCT	54	MDT vs. Observation	Rate of disease progression at 6 months	Disease progression was improved in MDT cohort (Progression at 6 months 19% vs. 61%, p=0.005)	No G3 or higher toxicities
Glicksman et al.	ORD	No limit	Single-arm Phase II Trial	37	PSMA-PET-guided MDT with SBRT or surgery, without ADT	Biochemical response	60% overall response rate with 22% having complete response	No G3 or higher toxicities
Kneebone et al.	ORD	1-3 nodal or bone metastases	Single-arm Phase II Clinical Trial	57	SBRT to metastatic sites without ADT	Biochemical failure***	At median follow up of 16 months, median bDFS was 11 months, with 31.9% bDFS at 15 months	No G3 or higher
Siva et al.	ORD	1-3 nodal or bone metastases	Feasibility Study	33	One fraction of SBRT to each lesion	Feasibility and tolerability	All but one patient completed the prescribed dose to metastatic sites	One patient with G3
Pezzulla et al.	OPD	≤ 5 non-visceral, nodal metastases	*Post hoc* analysis of phase I clinical trials	38	SBRT to lesions (in addition to concurrent ADT)	Biochemical response and toxicity	2-year next line systemic therapy-free survival of 67.7%	One patient with > G1

*Defined as not having visceral metastases or ≥4 bone metastases with at least one outside of the spine/pelvis.

**N=16 with prostate cancer.

***PSA level of nadir +0.2ng/mL following MDT.

RCT, randomized controlled trial; ADT, androgen deprivation therapy; OS, overall survival; OMD, oligometastatic disease; RT, radiotherapy; ORD, oligorecurrent disease; MDT, metastasis-directed therapy; SBRT, stereotactic body radiotherapy; OPD, oligoprogressive disease; G#, grade #.

HORRAD is another phase III study that examined the effect of local treatment to the prostate in the setting of *de novo* OPC. 432 men with previously untreated *de novo* mPCa, PSA>20 ng/mL and unlimited bone metastases were randomized to either ADT alone or ADT with RT to the primary tumor consisting of either 70 Gy in 35 fractions given over 7 weeks or 57.76 Gy in 19 fractions given three times per week. The primary endpoint, OS, was not significant between the two arms (HR=0.90, 95% CI 0.70-1.14). Moreover, subgroup analysis of patients with fewer than 5 metastases also did not demonstrate a statistically significant difference in OS (HR 0.68, 95% CI 0.42-1.10) (31). While this may cast doubt on the benefit of local therapy in the oligometastatic setting, it is imperative to note that a pooled analysis of the STAMPEDE and HORRAD trials showed a 7% improvement in 3-year survival in men with fewer than 5 bone metastases (32). The role of local therapy in the setting of metastatic disease is further being explored in the prospective trials PEACE-1 and SWOG 1802, although the eligibility for either trial include patients with any number of metastatic lesions, making their relevance in OPC uncertain at this time (33, 34).

The concept of treating both the primary tumor in addition to metastasis-directed therapy (MDT), or total consolidative therapy (TCT), is gaining traction in OPC. Most data regarding TCT comes from small, retrospective studies. For instance, one experience from the University of Rome consisting of 37 previously radiotherapy-naïve patients with ≤5 metastases who underwent TCT showed promising results, with OS and biochemical relapse-free survival (b-RFS) at 5 years of 65.4% and 39.3%, respectively, with no instances of ≥G3 acute or late toxicity reported (35). A separate retrospective study reported by Deantoni et al. included 39 men with bone-only (≤2) metastases showed similarly favorable outcomes with TCT, with 4-year rates of b-RFS and OS of 53.3% and 82.4%, respectively. In this study, no acute ≥G3 toxicities were noted, and no toxicity of any severity was reported for treatment of metastatic sites (36). The only prospective evidence for TCT in OPC comes from a single prospective registry trial that consisted of 12 men with *de novo* OPC (≤5 metastases) who underwent sequential treatment with neoadjuvant chemotherapy, radical prostatectomy, MDT+/- adjuvant RT to the prostatic bed/pelvis followed by adjuvant ADT. At 3 years, 67% of men were free from biochemical failure and all remained alive, with no ≥G3 acute toxicities and no late toxicity of any severity reported (37). An ongoing small single-arm phase II trial (NCT03298087) is also evaluating the efficacy of TCT in *de novo* OPC patients with prostatectomy, MDT to metastatic lesions, and adjuvant radiotherapy with 6-months of ADT, apalutamide and abiraterone, with final results not yet reported (**Table 2**) (38). Taken together, these studies suggest that TCT for men with *de novo* OPC may be a feasible management strategy with low risk of clinically significant toxicity.

**Table 2:** Ongoing/future trials evaluating radiotherapy/MDT in (oligo)metastatic prostate cancer.

Study	Disease Type	Metastatic Burden	Study Type	Randomization (if applicable)	Primary Outcome
STAMPEDE Arm M	*De novo* OMD	≤5 lesions	Phase III RCT	SOC (+ prostate RT/surgery) vs. SOC (+ prostate RT/surgery) + MDT	OS
NCT03298087	*De novo* OMD	≤5 lesions	Single-arm Phase II	Prostatectomy + MDT + adjuvant RT with 6 months of ADT/apalutamide/abiraterone	PSA <0.05ng/mL 6 months after recovery of testosterone
PLATON	*De novo* OMD and ORD	≤5 lesions	Phase III RCT	SOC* vs. SOC* + MDT	FFS at 6 years
NRG-GU011	ORD	≤5 lesions	Phase II RCT	MDT + placebo vs. MDT + relugolix	rPFS by conventional imaging
DART	ORD	≤5 lesions	Phase II RCT	MDT vs. MDT + darolutamide	MFS at 2 years by PET
RADIOSA	ORD	≤3 lesions	Phase II RCT	MDT vs. MDT + LHRH agonist/antagonist	PFS
ECOG-ACRIN 8191	Biochemical recurrence	No limit**	Phase III RCT	Salvage RT + ADT/apalutamide vs. Salvage RT + ADT/apalutamide + MDT	PFS, QOL
FORCE	OPD	≤5 lesions	Phase II RCT	SOC vs. SOC + SBRT to all sites of disease	Mean response duration
PEACE-1	*De novo* metastatic disease	No limit	Phase III RCT	SOC (ADT +/- docetaxol) vs. SOC + abiraterone vs. SOC + prostate RT vs. SOC + abiraterone + prostate RT	OS, rPFS

*SOC includes ablative therapy (surgery or SBRT) to prostate for patients with untreated prostate primary and low volume metastatic disease. **Conventional imaging negative, no limit on 18F-fluciclovine PET positive lesions.

OMD, oligometastatic disease; RCT, randomized controlled trial; SOC, standard of care; MDT, metastasis-directed therapy; OS, overall survival; ADT, androgen deprivation therapy; PSA, prostate-specific antigen; ORD, oligorecurrent disease; SBRT, stereotactic body radiotherapy; FFS, failure-free survival; rPFS, radiographic progression-free survival; MFS, metastasis-free survival; LHRH, luteinizing hormone-releasing hormone; PFS, progression-free survival; QOL, quality of life; OPD, oligoprogressive disease; RT, radiotherapy.

While the previously discussed studies offer promise regarding the potential for TCT in the setting of OPC, phase III trials remain the gold standard to evaluate the benefit of MDT in addition to local therapy to the prostate for men with OPC. One such future trial is STAMPEDE Arm M that will enroll men with *de novo* OPC who plan to undergo local therapy (surgery or RT) and will be randomized to receiving systemic therapy with or without MDT, with those receiving MDT effectively receiving TCT (**Table 2**). However, it is imperative to note that *de novo* OPC in STAMPEDE Arm M is being defined by CI only, rather than by more sensitive molecular imaging such as PSMA-PET, leaving the question of how to optimally manage men with *de novo* OPC defined by PSMA-PET unanswered (39). The ongoing phase III Canadian PLATON trial (NCT03784755) also defines *de novo* or recurrent OPC using CI and randomizes these patients to with or without MDT (40). Designing future trials to assess the efficacy of PET-guided MDT in *de novo* OPC is warranted to complement the results from these CI-defined OPC trials, especially with the recent rapid adoption of PSMA-PET for upfront initial staging.

## Radiotherapy in Oligorecurrent Disease (ORD)

Most evidence for MDT in oligometastatic disease comes from phase II RCTs in the setting of ORD, although the diversity of primary endpoints among studies can make the clinical application of RT unclear (see **Table 1**). One such trial is the SABR-COMET, in which 99 patients with ORD from various malignancies with 1-5 metastases underwent a 2:1 randomization to MDT with stereotactic radiotherapy vs. SOC for their respective malignancies, with a primary endpoint of OS. It is important to note that only 16 of the 99 patients included in this trial had prostate cancer. At 5 years, OS was 42.3% in the MDT arm compared to only 17.7% who were treated with SOC. While this appears to be an impressive improvement in OS with the addition of MDT, this study is not without criticisms. First, given that this study included multiple histologies, with prostate cancer representing only a small fraction, it is difficult to apply these results to all patients with oligorecurrent prostate cancer. Moreover, there was a skewed proportion of prostate cancer patients between the two arms, with prostate cancer patients comprising 21% of those who received MDT compared to only 6% of those who received SOC. The favorable natural history of prostate cancer may have led to a higher OS rate in the MDT arm. A final criticism of this study is that the rate of G5 toxicity was 4.5% in those treated with MDT, which is exceedingly high and not consistent with the plethora of evidence that supports the safety of MDT, although none of these deaths occurred in patients with prostate cancer. Of note, 2/3 of G5 toxicities occurred in patients undergoing thoracic SBRT, which is uncommon in the setting of mPCa (41, 42).

Many of the concerns regarding SABR-COMET and its relevance to men with prostate ORD can be addressed by having a more homogeneous study population. STOMP is a phase II RCT that randomized 62 men with prostate cancer who had asymptomatic ORD, defined as 3 or fewer metastases on choline-PET, after prior primary curative therapy in a 1:1 fashion to MDT or observation. The primary outcome of this study was ADT-free survival, with indications to start ADT for symptomatic or local progression or development of additional metastases. At a median follow up of 3 years, median ADT-free survival was 13 months in the surveillance cohort compared to 21 months for those who received MDT (HR 0.60, 80% CI 0.40-0.90, p=0.11). Of note, in contrast to the severe toxicities noted in SABR-COMET, no G2-5 toxicities were reported in this study (43). ORIOLE is another phase II RCT that utilized MDT in the oligorecurrent setting. This trial randomized 54 men with hormone-sensitive mPCa with ≤3 metastases based on CI in a 2:1 fashion to MDT with SBRT or observation. The study’s primary outcome was the rate of disease progression at 6 months, which was significantly improved in the MDT cohort compared to the observation group (19% vs. 61%, p=0.005). Again, in contrast to SABR-COMET, no G3 or greater toxicities were reported in this study (44).

Several smaller single-arm prospective studies have also demonstrated the safety and efficacy of MDT in ORD. A study by Glicksman et al. enrolled patients with rising PSA after radical prostatectomy and either adjuvant or salvage RT who had negative CI but positive PSMA-PET findings on restaging scans. Patients were treated with PSMA-PET-guided MDT with SBRT (n=27) or surgery (n=10) without ADT. At a median follow up of 15.9 months, 22% of treated men had an undetectable PSA, with a 60% overall response rate and a median time to PSA progression of 17.7 months, allowing for further delay in ADT administration. No G3 or greater toxicities were noted in patients who received SBRT (45). Similarly, a study by Kneebone et al. treated 57 oligorecurrent patients with 1-3 metastatic nodal or bone sites detected *via* PSMA-PET with SBRT to the metastatic sites without ADT. The primary endpoint was biochemical failure, defined as PSA level of nadir +0.2ng/mL following MDT. At a median follow-up of 16 months, the median biochemical disease-free survival (bDFS) was 11 months, with 31.9% bDFS at 15 months. No G3 or higher toxicities were noted in this study (46). A separate feasibility study by Siva et al. treating 33 patients with 1-3 metastases by NaF-PET and CI also showed favorable results with or without ADT, with all but one patient completing the prescribed 20 Gy in 1 fraction dose to sites of metastatic disease. Two-year distant PFS was 39%, and 48% of those treated without ADT remained free from ADT at 2 years. Only one G3 toxicity was reported (47).

The use of MDT in ORD is not only clinically beneficial but can also be a cost-effective treatment strategy. One cost-utility analysis based on the STOMP trial showed that MDT appeared to have an 85.9% probability of being cost-effective in comparison to surveillance with delayed ADT and a 100% probability of cost-effectiveness in comparison to immediate ADT (48). A separate study utilizing the SABR-COMET clinical data found that MDT was cost-effective in 97% of all iterations in comparison to standard of care on probabilistic sensitivity analysis. While SABR-COMET was based in Canada, an additional analysis was performed based on United States payer perspective and yielded similar results with a 98% probability of cost-effectiveness (49).

Taken as a whole, these studies evaluating the use of MDT in the setting of oligorecurrent prostate cancer demonstrate that MDT is a cost-effective treatment strategy associated with minimal toxicity and the potential to delay disease progression and the use of ADT/systemic therapy. Furthermore, for a subset of patients, albeit likely small, MDT for ORD may even achieve long-term disease control. However, prospective phase III studies are warranted to further investigate the clinical benefit of MDT in this setting. Currently, several phase II trials are ongoing to evaluate the potential benefit of combining a short-course of hormonal therapy with MDT to improve disease control (**Table 2**). NRG-GU011 (NCT05053152), DART (NCT04641078), and RADIOSA (NCT03940235) aim to investigate the addition of relugolix, darolutamide, and LHRH agonists/antagonists, respectively, to MDT (50–52). On the other hand, in the setting of biochemical recurrence after prostatectomy, phase III ECOG-ACRIN 8191 seeks to evaluate the role of MDT in patients with CI-negative but 18F-fluciclovine PET-positive extra-pelvic metastases at time of PSA progression, which addresses a timely question of whether local therapy of PET-detected metastatic disease (of lower tumor burden compared to CI-detected disease) will alter patient outcomes (**Table 2**) (53).

## Radiotherapy in (OPD) Oligoprogressive Disease

With the advent of systemic therapy options that have led to prolonged survival compared to historical standards, even in men with widespread metastatic disease, there has been growing interest in the use of radiotherapy for OPD, with the rationale being that treating sites of oligoprogression may allow patients to remain on the same agent for a longer duration by eradicating tumor clones that have developed resistance to the agent (11). However, no large prospective study exists on the clinical utility of radiotherapy in the setting of OPD, although a prospective trial is currently accruing (**Table 2**). The main source of prospective data regarding OPD is a pooled analysis of two phase I studies that assessed the use of stereotactic RT in primary, oligorecurrent and oligometastatic cancers. This analysis included men with metastatic castration-resistant prostate cancer (mCR-PCa) with 5 or fewer metastases (without visceral metastases) and progressive nodal metastases. In total, 38 patients were included, all of whom were receiving ADT at time of treatment. Two-year next line systemic therapy-free survival (NEST-FS) was 67.7% and only one patient had a >G1 toxicity (G2 dysphagia for supraclavicular field treatment) (54). Beyond this, one must look to retrospective studies for further data. Herein, a subset of these studies will be discussed. One retrospective study by Onal et al. reviewed 54 men with mCR-PCa with 5 or fewer PSMA-PET or bone scan-detected progressive lesions in the lymph nodes or bones treated with SBRT to all lesions while receiving abiraterone or enzalutamide. With a median follow-up of 19.1 months, median prostate cancer-specific survival (PCSS) and PFS were 27.8 months and 12.7 months, respectively. Of note, the number of oligoprogressive lesions requiring treatment and the time between start of abiraterone or enzalutamide and RT treatment were prognostic factors for PCSS on univariate analysis, although the number of lesions treated was only borderline significant on multivariate analysis (p=0.06). Further supporting the use of RT to delay a change in systemic therapy, SBRT to oligoprogressive lesions allowed for continuation of the patients’ current systemic therapies for a median of 8.6 months (11). A second retrospective study by Onal et al. of 67 patients treated with SBRT to 5 or fewer PSMA-positive oligoprogressive lesions showed similarly favorable results, with 2-year OS of 86.9% and only 32.8% of patients progressing to next-line systemic therapy at a median time of 16.4 months from completing SBRT (55).

Likewise, Deek et al. reported outcomes of 68 patients with mCR-PCa who received RT to 1-5 progressive lesions. Following MDT, median time to PSA recurrence, time to next intervention and distant metastasis-free survival were 9.67 months, 15.6 months and 10.8 months, respectively. Median OS had not been reached at median follow-up of 30.9 months. Of note, patients with consolidation of all disease (progressive and stable lesions) were also included in this study, with those receiving TCT having improved outcomes compared to those treated to oligoprogressive lesions alone (56). Additional retrospective studies have shown similar findings with MDT to oligoprogressive sites delaying the need to change systemic therapy, with reported median time to NEST-FS of 15.2 months (57), 16 months (58) and 21.8 months (59), and prolonged distant progression-free survival of 21.6% at 2-years (60).

While additional prospective evidence is needed to further clarify the role of RT in oligoprogressive prostate cancer, these retrospective studies demonstrating a prolongation of NEST-FS and/or PFS suggest that MDT to oligoprogressive sites of disease is a potential treatment strategy that may increase the effective time-window of any given systemic therapy for at least a subset of men with OPD. The phase II FORCE trial seeks to further explore this notion in a different light. Rather than examining NEST-FS or PFS without changing systemic therapy, the primary objective of FORCE trial is to assess the mean duration of response of men with oligometastatic castrate-resistant disease receiving next-line systemic therapy randomized to with or without MDT (61). Certainly, more prospective trials are necessary in the OPD setting to optimize the use of MDT to maximize the utility of systemic therapies available for castrate-resistant disease.

## Conclusion

While the role of RT in *de novo* OPC, ORD and OPD remains unclear, clinically meaningful outcomes have been demonstrated with MDT to OPD and ORD. Larger trials are needed to answer several questions, including which patients will not benefit from this strategy and which patients stand to receive the most benefit, perhaps even cure. With various ongoing studies in this realm currently underway (**Table 2**), the clinical benefit of MDT in the oligometastatic setting will likely be further clarified soon.

## Author Contributions

AY and S-JW contributed to the conception and outline of the manuscript design. AY and S-JW wrote the manuscript. AS, AY, and S-JW generated the tables. All authors contributed to manuscript revision, read, and approved the submitted version.

## Conflict of Interest

The authors declare that the research was conducted in the absence of any commercial or financial relationships that could be construed as a potential conflict of interest.

## Publisher’s Note

All claims expressed in this article are solely those of the authors and do not necessarily represent those of their affiliated organizations, or those of the publisher, the editors and the reviewers. Any product that may be evaluated in this article, or claim that may be made by its manufacturer, is not guaranteed or endorsed by the publisher.

## References

[1] Culp MB Soerjomataram I Efstathiou JA Bray F Jemal A. Recent Global Patterns in Prostate Cancer Incidence and Mortality Rates. Eur Urol (2020) 77(1):38–52. doi: 10.1016/j.eururo.2019.08.005 31493960

[2] Taitt HE. Global Trends and Prostate Cancer: A Review of Incidence, Detection, and Mortality as Influenced by Race, Ethnicity, and Geographic Location. Am J Mens Health (2018) 12(6):1807–23. doi: 10.1177/1557988318798279 PMC6199451 30203706

[3] Fletcher SA von Landenberg N Cole AP Gild P Choueiri TK Lipsitz SR. Contemporary National Trends in Prostate Cancer Risk Profile at Diagnosis. Prostate Cancer Prostatic Dis (2020) 23(1):81–7. doi: 10.1038/s41391-019-0157-y 31235801

[4] Kensler KH Pernar CH Mahal BA Nguyen PL Trinh QD Kibel AS. Racial and Ethnic Variation in PSA Testing and Prostate Cancer Incidence Following the 2012 USPSTF Recommendation. J Natl Cancer Inst (2021) 113(6):719–26. doi: 10.1093/jnci/djaa171 PMC8168268 33146392

[5] Hamdy FC Donovan JL Lane JA Mason M Metcalfe C Holding P. 10-Year Outcomes After Monitoring, Surgery, or Radiotherapy for Localized Prostate Cancer. N Engl J Med (2016) 375(15):1415–24. doi: 10.1056/NEJMoa1606220 27626136

[6] Moris L Cumberbatch MG Van den Broeck T Gandaglia G Fossati N Kelly B. Benefits and Risks of Primary Treatments for High-Risk Localized and Locally Advanced Prostate Cancer: An International Multidisciplinary Systematic Review. Eur Urol (2020) 77(5):614–27. doi: 10.1016/j.eururo.2020.01.033 32146018

[7] Teo MY Rathkopf DE Kantoff P. Treatment of Advanced Prostate Cancer. Annu Rev Med (2019) 70:479–99. doi: 10.1146/annurev-med-051517-011947 PMC6441973 30691365

[8] Catton CN Gospodarowicz MK. Palliative Radiotherapy in Prostate Cancer. Semin Urol Oncol (1997) 15(1):65–72. 9050141

[9] Radiotherapy to the Primary Tumour for Newly Diagnosed, Metastatic Prostate Cancer (STAMPEDE): A Randomised Controlled Phase 3 Trial - ScienceDirect . Available at: https://www.sciencedirect.com/science/article/pii/S0140673618324863?via%3Dihub (Accessed 2022 Mar 4). 10.1016/S0140-6736(18)32486-3 PMC6269599 30355464

[10] Rao A Vapiwala N Schaeffer EM Ryan CJ. Oligometastatic Prostate Cancer: A Shrinking Subset or an Opportunity for Cure? Am Soc Clin Oncol Educ Book (2019) 39):309–20. doi: 10.1200/EDBK_239041 31099652

[11] Onal C Kose F Ozyigit G Aksoy S Oymak E Muallaoglu S. Stereotactic Body Radiotherapy for Oligoprogressive Lesions in Metastatic Castration-Resistant Prostate Cancer Patients During Abiraterone/Enzalutamide Treatment. Prostate. (2021) 81(9):543–52. doi: 10.1002/pros.24132 33905131

[12] Foster CC Weichselbaum RR Pitroda SP. Oligometastatic Prostate Cancer: Reality or Figment of Imagination? Cancer (2019) 125(3):340–52. doi: 10.1002/cncr.31860 PMC6587458 30521067

[13] Larbi A Dallaudière B Pasoglou V Padhani A Michoux N Vande Berg BC. (WB-MRI) Assessment of Metastatic Spread in Prostate Cancer: Therapeutic Perspectives on Targeted Management of Oligometastatic Disease. Prostate. (2016) 76(11):1024–33. doi: 10.1002/pros.23196 27197649

[14] Müller PJ Dietlein M Kobe C Heidenreich A Drzezga A. Oligometastatic Disease in Biochemical Recurrence of Prostate Cancer: Prevalence on PSMA PET/CT and Consecutive Metastasis-Directed Therapy - Experience at a Tertiary Referral Center. Nukl Nucl Med (2022). doi: 10.1055/a-1697-8111 35388442

[15] Halsted WSI. The Results of Operations for the Cure of Cancer of the Breast Performed at the Johns Hopkins Hospital From June, 1889, to January, 1894. Ann Surg (1894) 20(5):497–555. doi: 10.1097/00000658-189407000-00075 PMC1493925 17860107

[16] Fisher B. Laboratory and Clinical Research in Breast Cancer–a Personal Adventure: The David A. Karnofsky Memorial Lecture. Cancer Res (1980) 40(11):3863–74. 7008932

[17] Hellman S. Karnofsky Memorial Lecture. Natural History of Small Breast Cancers. . J Clin Oncol Off J Am Soc Clin Oncol (1994) 12(10):2229–34. doi: 10.1200/JCO.1994.12.10.2229 7931493

[18] Deek MP van der Eecken K Phillips R Parikh NR Isaacsson Velho P Lotan TL. The Mutational Landscape of Metastatic Castration-Sensitive Prostate Cancer: The Spectrum Theory Revisited. Eur Urol (2021) 80(5):632–40. doi: 10.1016/j.eururo.2020.12.040 PMC10262980 33419682

[19] Tomlinson JS Jarnagin WR DeMatteo RP Fong Y Kornprat P Gonen M. Actual 10-Year Survival After Resection of Colorectal Liver Metastases Defines Cure. J Clin Oncol (2007) 25(29):4575–80. doi: 10.1200/JCO.2007.11.0833 17925551

[20] Gomez DR Tang C Zhang J Blumenschein GR Hernandez M Lee JJ. Local Consolidative Therapy Vs. Maintenance Therapy or Observation for Patients With Oligometastatic Non–Small-Cell Lung Cancer: Long-Term Results of a Multi-Institutional, Phase II, Randomized Study. J Clin Oncol (2019) 37(18):1558–65. doi: 10.1200/JCO.19.00201 PMC6599408 31067138

[21] Wang XS Bai YF Verma V Yu RL Tian W Ao R. Randomized Trial of First-Line Tyrosine Kinase Inhibitor With or Without Radiotherapy for Synchronous Oligometastatic EGFR-Mutated NSCLC. J Natl Cancer Inst (2022) djac015. doi: 10.1093/jnci/djac015 35094066 PMC10248839

[22] Trovo M Furlan C Polesel J Fiorica F Arcangeli S Giaj-Levra N. Radical Radiation Therapy for Oligometastatic Breast Cancer: Results of a Prospective Phase II Trial. Radiother Oncol J Eur Soc Ther Radiol Oncol (2018) 126(1):177–80. doi: 10.1016/j.radonc.2017.08.032 28943046

[23] Milano MT Katz AW Zhang H Huggins CF Aujla KS Okunieff P. Oligometastatic Breast Cancer Treated With Hypofractionated Stereotactic Radiotherapy: Some Patients Survive Longer Than a Decade. Radiother Oncol J Eur Soc Ther Radiol Oncol (2019) 131:45–51. doi: 10.1016/j.radonc.2018.11.022 30773186

[24] Franzese C Comito T Viganò L Pedicini V Franceschini D Clerici E. Liver Metastases-Directed Therapy in the Management of Oligometastatic Breast Cancer. Clin Breast Cancer. (2020) 20(6):480–6. doi: 10.1016/j.clbc.2020.05.006 32631769

[25] National Comprehensive Cancer Network. Prostate Cancer . Available at: https://www.nccn.org/professionals/physician_gls/pdf/prostate.pdf.

[26] Hofman MS Lawrentschuk N Francis RJ Tang C Vela I Thomas P. Prostate-Specific Membrane Antigen PET-CT in Patients With High-Risk Prostate Cancer Before Curative-Intent Surgery or Radiotherapy (proPSMA): A Prospective, Randomised, Multicentre Study. Lancet Lond Engl (2020) 395(10231):1208–16. doi: 10.1016/S0140-6736(20)30314-7 32209449

[27] Tsai CJ Yang JT Guttmann DM Shaverdian N Shepherd AF Eng J. Consolidative Use of Radiotherapy to Block (CURB) Oligoprogression ― Interim Analysis of the First Randomized Study of Stereotactic Body Radiotherapy in Patients With Oligoprogressive Metastatic Cancers of the Lung and Breast. Int J Radiat Oncol (2021) 111(5):1325–6. doi: 10.1016/j.ijrobp.2021.09.014

[28] Ali A Hoyle A Haran ÁM Brawley CD Cook A Amos C. Association of Bone Metastatic Burden With Survival Benefit From Prostate Radiotherapy in Patients With Newly Diagnosed Metastatic Prostate Cancer: A Secondary Analysis of a Randomized Clinical Trial. JAMA Oncol (2021) 7(4):555–63. doi: 10.1001/jamaoncol.2020.7857 PMC7893550 33599706

[29] Kyriakopoulos CE Chen YH Carducci MA Liu G Jarrard DF Hahn NM. Chemohormonal Therapy in Metastatic Hormone-Sensitive Prostate Cancer: Long-Term Survival Analysis of the Randomized Phase III E3805 CHAARTED Trial. J Clin Oncol Off J Am Soc Clin Oncol (2018) 36(11):1080–7. doi: 10.1200/JCO.2017.75.3657 PMC5891129 29384722

[30] Lester-Coll NH Ades S Yu JB Atherly A Wallace HJ Sprague BL. Cost-Effectiveness of Prostate Radiation Therapy for Men With Newly Diagnosed Low-Burden Metastatic Prostate Cancer. JAMA Netw Open (2021) 4(1):e2033787. doi: 10.1001/jamanetworkopen.2020.33787 33439266 PMC7807293

[31] Boevé LMS Hulshof MCCM Vis AN Zwinderman AH Twisk JWR Witjes WPJ. Effect on Survival of Androgen Deprivation Therapy Alone Compared to Androgen Deprivation Therapy Combined With Concurrent Radiation Therapy to the Prostate in Patients With Primary Bone Metastatic Prostate Cancer in a Prospective Randomised Clinical Trial: Data From the HORRAD Trial. Eur Urol (2019) 75(3):410–8. doi: 10.1016/j.eururo.2018.09.008 30266309

[32] Burdett S Boevé LM Ingleby FC Fisher DJ Rydzewska LH Vale CL. Prostate Radiotherapy for Metastatic Hormone-Sensitive Prostate Cancer: A STOPCAP Systematic Review and Meta-Analysis. Eur Urol (2019) 76(1):115–24. doi: 10.1016/j.eururo.2019.02.003 PMC6575150 30826218

[33] S1802 SWOG . Available at: https://www.swog.org/clinical-trials/s1802 (Accessed 2022 Mar 29).

[34] UNICANCER. A Prospective Randomised Phase III Study Of Androgen Deprivation Therapy With Or Without Docetaxel With Or Without Local Radiotherapy With Or Without Abiraterone Acetate And Prednisone In Patients With Metastatic Hormone-Naïve Prostate Cancer (2021). Available at: https://clinicaltrials.gov/ct2/show/NCT01957436 (Accessed 2022 Mar 28).

[35] Reverberi C Massaro M Osti MF Anzellini D Marinelli L Montalto A. Local and Metastatic Curative Radiotherapy in Patients With *De Novo* Oligometastatic Prostate Cancer. Sci Rep (2020) 10(1):17471. doi: 10.1038/s41598-020-74562-3 33060732 PMC7563994

[36] Deantoni CL Fodor A Cozzarini C Fiorino C Brombin C Di Serio C. Prostate Cancer With Low Burden Skeletal Disease at Diagnosis: Outcome of Concomitant Radiotherapy on Primary Tumor and Metastases. Br J Radiol (2020) 93(1108):20190353. doi: 10.1259/bjr.20190353 31971828 PMC7362924

[37] Reyes DK Rowe SP Schaeffer EM Allaf ME Ross AE Pavlovich CP. Multidisciplinary Total Eradication Therapy (TET) in Men With Newly Diagnosed Oligometastatic Prostate Cancer. Med Oncol Northwood Lond Engl (2020) 37(7):60. doi: 10.1007/s12032-020-01385-7 PMC7286864 32524295

[38] VA Office of Research and Development. Systemic and Tumor-Directed Therapy for Oligometastatic Prostate Cancer (2022). Available at: https://clinicaltrials.gov/ct2/show/NCT03298087 (Accessed 2022 Apr 18).

[39] Preisser F Chun F Banek S Wenzel M Graefen M Steuber T. Management and Treatment Options for Patients With *De Novo* and Recurrent Hormone-Sensitive Oligometastatic Prostate Cancer. Prostate Int (2021) 9(3):113–8. doi: 10.1016/j.prnil.2020.12.003 PMC8498729 34692582

[40] Canadian Cancer Trials Group. A Randomized Phase III Trial of Local Ablative Therapy For Hormone Sensitive Oligometastatic Prostate Cancer [PLATON] (2022). Available at: https://clinicaltrials.gov/ct2/show/NCT03784755 (Accessed 2022 Apr 18).

[41] Palma DA Olson R Harrow S Gaede S Louie AV Haasbeek C. Stereotactic Ablative Radiotherapy for the Comprehensive Treatment of Oligometastatic Cancers: Long-Term Results of the SABR-COMET Phase II Randomized Trial. J Clin Oncol Off J Am Soc Clin Oncol (2020) 38(25):2830–8. doi: 10.1200/JCO.20.00818 PMC7460150 32484754

[42] Sogono P Ball DL Siva S. SABR-COMET: A New Paradigm of Care Lights Up the Twilight of Metastatic Disease. Ann Transl Med (2019) 7(22):615–5. doi: 10.21037/atm.2019.10.96 PMC6944625 31930016

[43] Ost P Reynders D Decaestecker K Fonteyne V Lumen N De Bruycker A. Surveillance or Metastasis-Directed Therapy for Oligometastatic Prostate Cancer Recurrence: A Prospective, Randomized, Multicenter Phase II Trial. J Clin Oncol (2018) 36(5):446–53. doi: 10.1200/JCO.2017.75.4853 29240541

[44] Phillips R Shi WY Deek M Radwan N Lim SJ Antonarakis ES. Outcomes of Observation vs Stereotactic Ablative Radiation for Oligometastatic Prostate Cancer: The ORIOLE Phase 2 Randomized Clinical Trial. JAMA Oncol (2020) 6(5):650–9. doi: 10.1001/jamaoncol.2020.0147 PMC7225913 32215577

[45] Glicksman RM Metser U Vines D Valliant J Liu Z Chung PW. Curative-Intent Metastasis-Directed Therapies for Molecularly-Defined Oligorecurrent Prostate Cancer: A Prospective Phase II Trial Testing the Oligometastasis Hypothesis. Eur Urol (2021) 80(3):374–82. doi: 10.1016/j.eururo.2021.02.031 33685838

[46] Kneebone A Hruby G Ainsworth H Byrne K Brown C Guo L. Stereotactic Body Radiotherapy for Oligometastatic Prostate Cancer Detected *via* Prostate-Specific Membrane Antigen Positron Emission Tomography. Eur Urol Oncol (2018) 1(6):531–7. doi: 10.1016/j.euo.2018.04.017 31158100

[47] Siva S Bressel M Murphy DG Shaw M Chander S Violet J. Stereotactic Abative Body Radiotherapy (SABR) for Oligometastatic Prostate Cancer: A Prospective Clinical Trial. Eur Urol (2018) 74(4):455–62. doi: 10.1016/j.eururo.2018.06.004 30227924

[48] De Bleser E Willems R Decaestecker K Annemans L De Bruycker A Fonteyne V. A Trial-Based Cost-Utility Analysis of Metastasis-Directed Therapy for Oligorecurrent Prostate Cancer. Cancers (2020) 12(1):E132. doi: 10.3390/cancers12010132 31947974 PMC7016808

[49] Qu XM Chen Y Zaric GS Senan S Olson RA Harrow S. Is SABR Cost-Effective in Oligometastatic Cancer? An Economic Analysis of the SABR-COMET Randomized Trial. Int J Radiat Oncol Biol Phys (2021) 109(5):1176–84. doi: 10.1016/j.ijrobp.2020.12.001 33309977

[50] Phase A II Double-Blinded. Placebo Controlled Trial of Prostate Oligometastatic Radiotherapy With or Without Androgen Deprivation Therapy in Oligometastatic Prostate Cancer (NRG Promethean) . Available at: https://www.nrgoncology.org/Clinical-Trials/Protocol/nrg-gu011?filter=nrg-gu011 (Accessed 2022 Apr 13).

[51] University Hospital. Ghent. Stereotactic Body Radiotherapy With or Without Darolutamide for OligoRecurrent Prostate Cancer: A Randomized Phase II Trial (DART) (2021). Available at: https://clinicaltrials.gov/ct2/show/NCT04641078 (Accessed 2022 Apr 18).

[52] European Institute of Oncology. Radioablation +/- Hormonotherapy for Prostate Cancer Oligorecurrences (RADIOSA Trial): Potential of Imaging and Biology (2021). Available at: https://clinicaltrials.gov/ct2/show/NCT03940235 (Accessed 2022 Apr 18).

[53] Cancer Research Group ECOG-ACRIN. Phase III Study of Local or Systemic Therapy INtensification DIrected by PET in Prostate CAncer Patients With Post-ProstaTEctomy Biochemical Recurrence (INDICATE) . Available at: https://clinicaltrials.gov/ct2/show/NCT04423211 (Accessed 2022 Apr 12).

[54] Pezzulla D Macchia G Cilla S Buwenge M Ferro M Bonome P. Stereotactic Body Radiotherapy to Lymph Nodes in Oligoprogressive Castration-Resistant Prostate Cancer Patients: A *Post Hoc* Analysis From Two Phase I Clinical Trials. Clin Exp Metastasis (2021) 38(6):519–26. doi: 10.1007/s10585-021-10126-7 34651242

[55] Onal C Ozyigit G Oymak E Guler OC Tilki B Hurmuz P. Stereotactic Radiotherapy to Oligoprogressive Lesions Detected With 68Ga-PSMA-PET/CT in Castration-Resistant Prostate Cancer Patients. Eur J Nucl Med Mol Imaging (2021) 48(11):3683–92. doi: 10.1007/s00259-021-05298-z 33693965

[56] Deek MP Taparra K Phillips R Velho PI Gao RW Deville C. Metastasis-Directed Therapy Prolongs Efficacy of Systemic Therapy and Improves Clinical Outcomes in Oligoprogressive Castration-Resistant Prostate Cancer. Eur Urol Oncol (2021) 4(3):447–55. doi: 10.1016/j.euo.2020.05.004 PMC7788526 32536574

[57] Franzese C Perrino M Marzo MA Badalamenti M Baldaccini D D’Agostino G. Oligoprogressive Castration-Resistant Prostate Cancer Treated With Metastases-Directed Stereotactic Body Radiation Therapy: Predictive Factors for Patients’ Selection. Clin Exp Metastasis (2022) 39(3):449–57. doi: 10.1007/s10585-022-10158-7 35190933

[58] Berghen C Joniau S Ost P Poels K Everaerts W Decaestecker K. Progression-Directed Therapy for Oligoprogression in Castration-Refractory Prostate Cancer. Eur Urol Oncol (2021) 4(2):305–9. doi: 10.1016/j.euo.2019.08.012 31558422

[59] Triggiani L Mazzola R Magrini SM Ingrosso G Borghetti P Trippa F. Metastasis-Directed Stereotactic Radiotherapy for Oligoprogressive Castration-Resistant Prostate Cancer: A Multicenter Study. World J Urol (2019) 37(12):2631–7. doi: 10.1007/s00345-019-02717-7 30859273

[60] Triggiani L Alongi F Buglione M Detti B Santoni R Bruni A. Efficacy of Stereotactic Body Radiotherapy in Oligorecurrent and in Oligoprogressive Prostate Cancer: New Evidence From a Multicentric Study. Br J Cancer. (2017) 116(12):1520–5. doi: 10.1038/bjc.2017.103 PMC5518848 28449007

[61] University of Michigan Rogel Cancer Center. FOcal Radiation for Oligometastatic Castration-Resistant Prostate Cancer (FORCE): A Phase II Randomized Trial (2021). Available at: https://clinicaltrials.gov/ct2/show/NCT03556904 (Accessed 2022 Apr 12).

